# Supplementary material for: The Effect of Probiotics on Health Outcomes in the Elderly: A Systematic Review of Randomized, Placebo-Controlled Studies
Source: Microorganisms. 2021 Jun 21;9(6):1344. doi: 10.3390/microorganisms9061344 (PMC8234958; doi:10.3390/microorganisms9061344)
Supplement: Supplementary file 1 [file microorganisms-09-01344-s001.zip › microorganisms-1271831-supplementary.pdf]

Supplemental Material S1- Complete search strategy

Pubmed

Search terms		
Probiotics		
	1	(("Probiotics"[Mesh]) OR (probiotic*[Title/Abstract]
Limits: RCT, English 2019- current, filter: aged 65+		
	1	

Embase

Search terms		
Probiotics		
	1	'probiotic agent'/exp OR probiotic*:ti,ab,kw
Limits: RCT, English 2019- current, filter: aged 65+, excluded conferencs abstracts		

Cochrane, Trials database

Search terms		
Probiotics		
	1	(MeSH descriptor: [Probiotics] explode all trees) OR (probiotic*):ti,ab,kw
	2	(MeSH descriptor: [Aged] explode all trees) OR (aged or elderly or old adult*):ti,ab,kw
	3	1 AND 2
Limits: RCT, 2019- current, excluded conferencs abstracts, ongoing studies CT.gov, WHO		
	3	

Cinahl

Search terms		
Probiotics		
	1	(MH "Probiotics") OR (TI probiotic* OR AB probiotic*)
Limits: RCT, English 2019- current, filter: aged 65+		

Scopus

Search terms		
Probiotics		
	1	(TITLE-ABS-KEY (probiotic*) AND TITLE-ABS-KEY ("randomized controlled trial*") AND TITLE-ABS-

		KEY (aged OR eldery)) AND PUBYEAR 2019- AND (LIMIT-TO (LANGUAGE , "English")) AND (LIMIT-TO (DOCTYPE , "artiklar"))

Supplementary Material S2- Quality and risk of bias assessment

	Moro-Garcia	Nyangale	Ostlund-Lagerstrom	Ouwehand	Shinkai	Spaiser	Kim	Ahmed	Arunachalam	Costabile	Gohel	Guilemard	Inoue	Macfarlane	Manzoni	Bartosch	Finamore	
**1. Study design**																		
1.1 Are the research question(s) and aim(s) clearly described?	3	3	3	3	3	3	3	3	3	3	3	3	3	3	3	3	3	
1.2 Were the following aspects of the study design and procedure described clearly?:																		
1.2.1 Inclusion and exclusion criteria	3	3	3	3	3	3	3	3	3	3	3	3	3	3	3	3	3	
1.2.2 Sample collection (time, procedure)	3	3	3	3	3	3	3	3	3	3	3	3	3	3	3	3	3	
Mean scoring (excl. NA):	3	3	3	3	3	3	3	3	3	3	3	3	3	3	3	3	3	
**2 Methods**																		
2.1 Were the methodological aspects described clearly?	3	3	3	3	3	3	3	3	3	3	3	3	3	3	3	3	3	
2.2 Are the recruitment procedure similar for all participants?	2	3	3	3	3	2	2	3	3	3	3	3	3	3	3	3	3	
2.3 Were the blinding procedure clearly described?	3	3	3	3	3	3	3	2	2	2	3	3	3	3	3	3	2	
2.4 Were the treatment allocation clearly described?	3	3	3	3	3	3	3	2	2	2	3	3	3	3	3	3	2	
Mean scoring (excl. NA):	2,75	3	3	3	3	2,75	2,75	2,5	2,5	2,5	3	3		3	3	3	2,5	
**3 Statistics**													3					
3.1 Were any power/sample size calculations reported?	0	0	3	0	3	3	3	0	0	2	0	3	3	3	0	0	0	
3.2 Are the statistical methods adequately described?	3	3	3	3	3	3	3	3	3	3	3	3	3	3	2	3	3	
3.3 Are the statistical methods relevant?	3	3	3	3	3	3	3	3	3	3	3	3	3	3	3	3	3	
Mean scoring (excl. NA):	2	2	3	2	3	3	3	2	2	2,67	2	3		3	1,67	2	2	
**4 Results**													3					
4.1 Are the results presented clearly in the text, figures and tables?	3	3	3	3	3	3	3	3	3	3	3	3	3	3	3	3	3	
4.4 Inclusion of supplementary material if relevant	NA	3	NA	NA	NA	NA	3	NA	NA	NA	NA	NA	3	3	NA	NA	NA	
Mean scoring (excl. NA):	3	3	3	3	3	3	3	3	3	3	3	3	3	3	3	3	3	
Mean of all items scored (excl. all NA):	**2,65**	**2,75**	**3**	**2,7333333**	**3**	**2,91667**	**2,922**	2,625	2,625	2,7925	2,75	3	3	3	2,6675	2,75	2,625	2,812169

Scoring:

3- Indicates that for that particular aspect of study design, the study has been designed or conducted in such a way as to minimize the risk of bias

2- Indicates that either the answer to the checklist question is not clear from the way the study is reported, or that the study may not have addressed all potential sources of bias for that particular aspect of study design

1- Should be reserved for those aspects of the study design in which significant sources of bias may persist

0- Should be reserved for those aspects in which the study under review fails to report how they have (or might have) been considered

NA- Should be reserved for those study design aspects that are not applicable given the study design under review
